# *Staphylococcus aureus* α-Toxin Triggers the Synthesis of B-Cell Lymphoma 3 by Human Platelets

**DOI:** 10.3390/toxins3020120

**Published:** 2011-01-28

**Authors:** Sebastian Schubert, Hansjörg Schwertz, Andrew S. Weyrich, Zechariah G. Franks, Stephan Lindemann, Monika Otto, Hagen Behr, Harald Loppnow, Axel Schlitt, Martin Russ, Peter Presek, Karl Werdan, Michael Buerke

**Affiliations:** 1 Department of Medicine III, Martin Luther University, Halle, Saale, Germany; Email: schubert-sebastian@gmx.net (S.S.); monika.otto@medizin.uni-halle.de (M.O.); hagen.behr@medizin.uni-halle.de (H.B.); harald.Loppnow@medizin.uni-halle.de (H.L.); axel.schlitt@medizin.uni-halle.de (A.S.); martin.russ@medizin.uni-halle.de (M.R.); karl.werdan@medizin.uni-halle.de (K.W.); 2 Program in Molecular Medicine, University of Utah, Salt Lake City, UT 84112, USA; Email: hansjorg.schwertz@u2m2.utah.edu (H.S.); andy.weyrich@u2m2.utah.edu (A.S.W.); zfranks@u2m2.utah.edu(Z.G.F.); 3 Department of Medicine III, Eberhard Karls University, Tübingen, Germany; Email: stephan.lindemann@med.uni-tuebingen.de; 4 Clinical Pharmacology, Martin Luther University, Halle, Saale, Germany; Email: peter.presek@medizin.uni-halle.de

**Keywords:** α-toxin, Bcl-3, protein synthesis

## Abstract

The frequency and severity of bacteremic infections has increased over the last decade and bacterial endovascular infections (*i.e.*, sepsis or endocarditis) are associated with high morbidity and mortality. Bacteria or secreted bacterial products modulate platelet function and, as a result, affect platelet accumulation at sites of vascular infection and inflammation. However, whether bacterial products regulate synthetic events in platelets is not known. In the present study, we determined if prolonged contact with staphylococcal α-toxin signals platelets to synthesize B-cell lymphoma (Bcl-3), a protein that regulates clot retraction in murine and human platelets. We show that α-toxin induced α_IIb_β_3_-dependent aggregation (EC_50_ 2.98 µg/mL ± 0.64 µg/mL) and, over time, significantly altered platelet morphology and stimulated *de novo* accumulation of Bcl-3 protein in platelets. Adherence to collagen or fibrinogen also increased the expression of Bcl-3 protein by platelets. α-toxin altered Bcl-3 protein expression patterns in platelets adherent to collagen, but not fibrinogen. Pretreatment of platelets with inhibitors of protein synthesis or the mammalian Target of Rapamycin (mTOR) decreased Bcl-3 protein expression in α-toxin stimulated platelets. In conclusion, *Staphylococcus* *aureus*-derived α-toxin, a pore forming exotoxin, exerts immediate (*i.e.*, aggregation) and prolonged (*i.e.*, protein synthesis) responses in platelets, which may contribute to increased thrombotic events associated with gram-positive sepsis or endocarditis.

## 1. Introduction

Mammalian platelets adhere to exposed subendothelial matrix, secrete granule contents, form multicellular aggregates, and serve as a *nidus* for plasma coagulation reactions [1]. Although these roles are well-defined in vascular injury, platelets also rapidly accumulate at sites of vascular injury and infection, including infective endocarditis and mycotic aneurysms [2,3]. In these types of situations, platelets frequently attach to vascular stents and valves where they serve as binding foci for bacteria and facilitate the recruitment of additional platelets and leukocytes to the infected site [2,3,4]. 

It is well known that agonists present in the infectious milieu, such as platelet activating factor (PAF) and thrombin, induce platelet aggregation and secretion [5]. However, bacterial toxins also modulate platelet reactivity. Among these, staphylococcal-derived α-toxin directly [6,7] or indirectly [8] activates platelets, the latter via generation of PAF by endothelial cells. α-toxin is a ~34 kDa protein secreted by *Staphylococcus aureus* (*S. aureus*) that binds and forms pores in target cell membrane [9]. The secretogogue and cytolytic activities of α-toxin target a number of mammalian cell types including leukocytes, endothelial cells, erythrocytes and platelets [9]. In addition, staphylococcal α-toxin is an important virulence factor in the pathogenesis of infections such as endocarditis or sepsis [3,6]. 

Although several studies have shown that α-toxin induces immediate activation responses in platelets (*i.e.*, within minutes) [6,7] , there is a growing appreciation that activated platelets continue to function over hours [10]. One prolonged function of platelets is *de novo* synthesis of proteins, including B-cell lymphoma (Bcl-3) [11,12,13,14]. Newly synthesized Bcl-3 binds Fyn and regulates cytoskeletal events in platelets [14] and Bcl-3 deficient platelets lack the capacity to retract fibrin-rich clots [13]. 

Because platelets, fibrin, and microcolonies of bacteria often vegetate with one another for extended periods of time in infective endocarditis and other infectious syndromes, we asked if bacterial toxins induce prolonged activation events in platelets. Specifically, we focused on the activating properties of α-toxin because *S. aureus* binds platelets [15] and is a common cause of infective endocarditis [6,16]. Our studies demonstrate that α-toxin induces platelets to aggregate, display morphologic features of activation, and synthesize Bcl-3 protein. 

## 2. Materials and Methods

### 2.1. Platelet Isolation

Research was approved by the University of Utah Institutional Review Board and by the Ethics Committee at Martin Luther University and all human participants gave written informed consent to participate in the study. Washed platelets were isolated as previously described [17]. The platelets were resuspended in Medium 199 (serum free) at a concentration (2 × 10^8^/mL) that falls within the normal range of platelets found in 1 mL of whole blood. For studies where the platelets were left in suspension, soluble fibrinogen (100 μg/mL) was added to the culture to mimic the presence of fibrinogen found in human plasma [13]. The purity and activation state of the platelet preparation was analyzed by FACS analysis with CD61, CD62, and CD154. On average, we observed less than two leukocytes per 1,000 platelets (data not shown). For the majority of experiments, the platelets were processed immediately (*i.e.*, time 0) or activated with 0.05 U/mL of thrombin (Sigma, Steinheim, Germany) or 500 ng/mL of α-toxin (Sigma) for designated times. This concentration of α-toxin approximates 50% of the amount released by 9 × 10^4^ CFUs of a clinical strain of *S. aureus* that was resuspended in 1 mL of M199 medium (Supplemental Figure 1). Using strains of *S. aureus* that produce α-toxin, Bayer *et al.* [6] observed that challenge inocula between 10^4^ and 10^6^ CFUs adhere to sterile cardiac vegetations and induce experimental endocarditis. 

The activation studies were done in platelets that were left in suspension or adhered to immobilized fibrinogen or collagen (see below). To gauge protein synthesis, platelets were pre-treated for 30 minutes with puromycin (Sigma), rapamycin (Calbiochem, Merck KGaA, Darmstadt, Germany), wortmannin (Sigma), or their vehicle (dimethylsulfoxide) (Sigma). 

### 2.2. Platelet Aggregation

Washed platelets were resuspended (2 × 10^8^ platelets/mL) in platelet poor plasma (PPP) and aggregation was measured with an APACT (Automated Platelet Aggregation Coagulation Tracer, Achrensburg, Germany) according to the method of Born [18]. Aggregation was induced by increasing concentrations of α-toxin. Abciximab, an antibody that blocks α_IIb_β_3_-dependent aggregation, was also tested against 5 μg/mL of α-toxin. This concentration was chosen based on the EC_50_ of α-toxin for inducing platelet aggregation. 

### 2.3. Adherence of Platelets to Immobilized Surfaces

Platelet adhesion was carried out in six-well plates or borosilicate chamber slides that were previously coated overnight (4 °C) with fibrinogen purified from human plasma (Calbiochem, Merck KGaA, Darmstadt, Germany), type I collagen (Sigma), or human serum albumin (HSA; Bayer, Leverkusen, Germany) as previously described by our group [12,19]. Before the start of each experiment, the coated wells were blocked with HSA (1%) and washed prior to adding platelets. Washed platelets were allowed to adhere to each surface in the presence of agonists (*i.e.*, α-toxin or thrombin) for the indicated times. At the end of each experiment, the platelets were placed directly in SDS-PAGE reducing buffer for Western analysis. 

### 2.4. Immunocytochemistry (ICC)

Platelets were left quiescent or activated as described above. Suspension cells were directly fixed in suspension using 2% paraformaldehyde for 20 minutes at room temperature and then adhered to vectabond^TM^ (Vector Laboratories, Burlingame, CA, U.S.A) coated glass-coverslips using a cytospin centrifuge (Shandon Cytospin, Thermo Fisher Scientific, Waltham, MA) as previously described [20,21]. Adherent platelets were directly fixed on borosilicate chamber slides [21]. The cells were stained with Alexa Fluo^®^ 488 phalloidin (A12379; Invitrogen, Eugene, OR), a high-affinity probe for F-actin, and Alexa Fluor^®^ 555 conjugate of wheat germ agglutinin (WGA; Invitrogen), a probe that binds sialic acids. The microscopy was performed using an Olympus IX81, FV300 (Olympus, Melville, NY, U.S.A) confocal-scanning microscope equipped with a 60×/1.42 NA oil objective for viewing platelets. An Olympus FVS-PSU/IX2-UCB camera and scanning unit and Olympus Fluoview FV 300 image acquisition software version 5.0 were used for recording. The images were further analyzed using Adobe Photoshop CS version 8.0 and ImageJ (NIH).

### 2.5. Western Blot Analysis

Platelet proteins were separated by electrophoresis using a 9% SDS-polyacrylamide gel. Proteins were transferred to a polyvinylidene disulfide membrane and probed for Bcl-3 as previously described [11,12,14]. The antibody directed against Bcl-3 was raised against amino acids 301–446 of human Bcl-3 (Santa Cruz Technology, Santa Cruz, CA, USA). Each sample was also probed for actin to ensure equal loading of proteins. 

### 2.6. Reverse Transcriptase–PCR (RT-PCR)

Total RNA was isolated from purified human platelets (1 × 10^9^) using 1 mL TRIzol^®^ reagent as previously described [11,14]. One μg of the RNA was used as a template for single-strand cDNA synthesis, which was generated with Moloney murine leukemia virus reverse transcriptase (GIBCO/BRL) using the conditions supplied by the manufacturer. The primers for Bcl-3 were the following: sense, 5’-CGA CGC AGT GGA CAT TAA GA-3’ and anti-sense, 5’-AGA TGG GGA AGG AAG GAA GA-3’. 

## 3. Results

First, we determined if α-toxin induces aggregation, an activity of platelets that occurs within seconds to minutes. We found that α-toxin induced platelet aggregation within a minute and in a concentration-dependent manner [Figure 1(A)]. The EC_50_ for α-toxin induced aggregation was 2.98 ± 0.64 µg/mL. Pretreatment of platelets with a blocking antibody directed against integrin α_IIb_β_3_ inhibited α-toxin induced platelet aggregation [Figure 1(B)]. 

**Figure 1:**
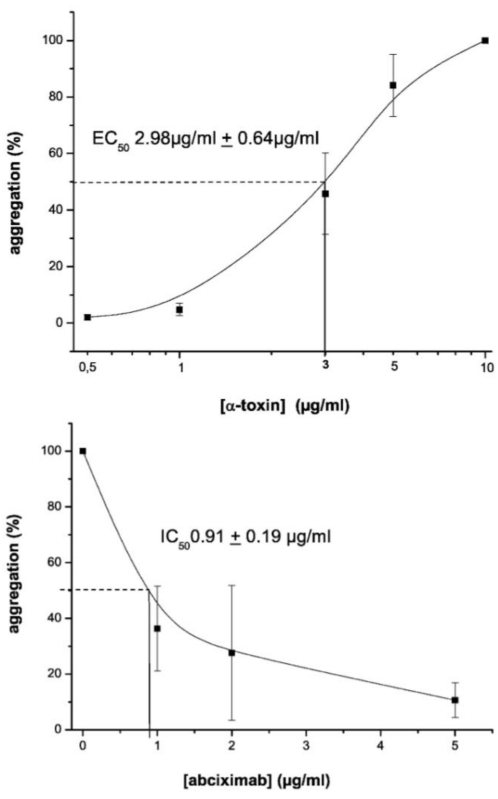
α-toxin induces integrin α_IIb_β_3_-dependent aggregation in platelets. Platelets were prepared as described in Materials and Methods and aggregation responses were monitored. (**A**) (upper) Aggregation was measured in platelets that were treated with increasing concentrations of α-toxin (0.5–10 μg/mL). (**B**) (lower) Aggregation was measured in platelets that were pretreated with varying concentrations of abciximab, which blocks α_IIb_β_3_-dependent aggregation, followed by stimulation with α-toxin (5 μg/mL). The fitted curves in both graphs are based on the mean ± SEM of four independent experiments.

Next, we assessed the effects of prolonged incubation of α-toxin on platelet morphology and Bcl-3 synthesis. Freshly-isolated (Figure 2(A), top panels) or unstimulated platelets that were cultured in suspension (Figure 2(A), middle panels) were discoid and their granules, as indexed by WGA, were evenly distributed throughout the cell indicative of a non-activated state. In contrast, α-toxin induced several distinct morphologic features in platelets after four hours (Figure 2(A), bottom panels): first, several platelets were visibly lysed and small, cellular fragments were readily observed in the extracellular milieu; second, intracellular granules were less obvious; third, pseudopodia extended from intact platelets and thin strands, which stained positive for WGA, interconnected platelets with one another; and fourth, polymerized actin coalesced to the cell center and was more condensed in α-toxin treated samples compared to unactivated controls. 

**Figure 2:**
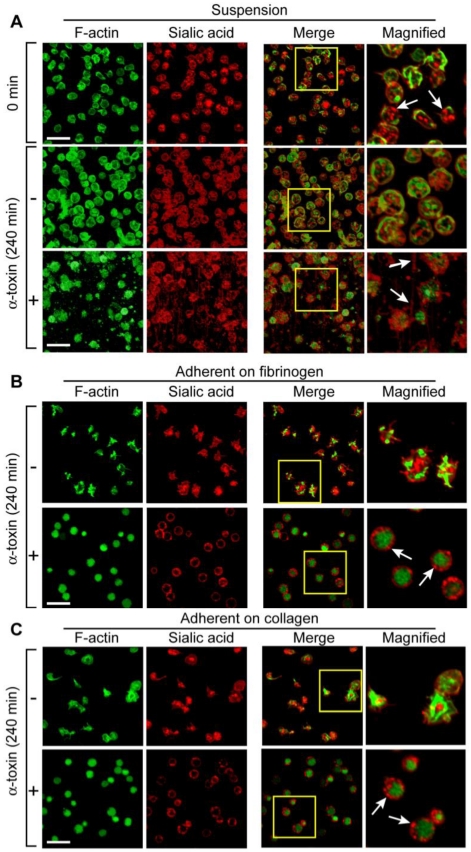
Prolonged exposure to α-toxin induces distinct morphologic changes in platelets. (**A**) Freshly-isolated platelets were fixed immediately (time = 0) or cultured for 240 minutes in suspension in the presence of soluble fibrinogen, with or without α-toxin, prior to fixation. (B-C) Platelets were adhered to immobilized fibrinogen (**B**) or collagen (**C**) in the presence or absence of α-toxin for 240 minutes. After this time period, the platelets were fixed. In (A-C), polymerized actin (F-actin) is represented by green while WGA staining (Sialic acid) is shown in red. The yellow represents co-localization of polymerized actin with sialic acids. The far right panels are high magnification insets taken from the boxes depicted in the “Merge” column. The white arrows point to discrete morphologic features. The scale bars equal 10 μm. These figures are representative of two independent experiments.

As expected, platelets adhered to fibrinogen or collagen over a four hour time period (Figures 2(B,C), top panels). The adherent platelets displayed features of activation, including pseudopodia extensions and their granules moved to the middle of the cell. Co-incubation with α-toxin did not significantly alter the adherence of platelets to either extracellular matrix (Figures 2(B,C), bottom panels). However, α-toxin stimulated platelets failed to extend pseudopodia and they remained round. They also distributed their remaining granules towards the cell membrane and condensed their polymerized actin [Figures 2(B,C), bottom panels]. 

**Figure 3:**
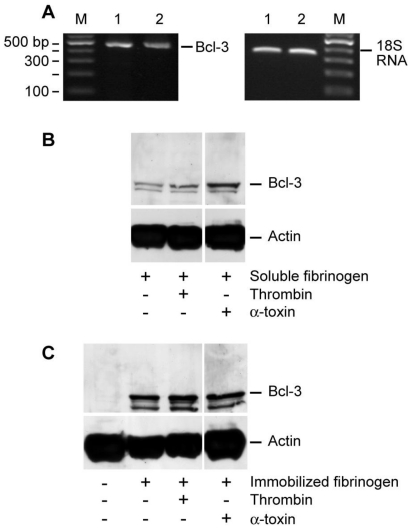
α-toxin accentuates Bcl-3 protein accumulation in platelets. (**A**) Freshly-isolated platelets were cultured in suspension in the presence of soluble fibrinogen alone (lane 1) or soluble fibrinogen and α-toxin (lane 2). After 240 minutes, Bcl-3 mRNA was assessed in both samples (right panel). The left panel displays 18S rRNA, as a loading control for Bcl-3. RNA gels are representative of three independent experiments. (**B**) Freshly-isolated platelets were cultured in suspension in the presence (lane 1) of soluble fibrinogen for 240 minutes and Bcl-3 (top panel) or actin (bottom panel) protein expression was assessed. Samples with soluble fibrinogen were also treated with thrombin (lane 2) or α-toxin (lane 3). (**C**) Freshly-isolated platelets were cultured in suspension (lane 1) or adhered to immobilized fibrinogen for 240 minutes and Bcl-3 (top panel) or actin (bottom panel) protein expression was assessed. Platelets that adhered to immobilized fibrinogen were treated with vehicle (lane 2), thrombin (lane 3), or α-toxin (lane 4). The protein gels (B-C) are representative of five independent experiments. The dividing lines between lanes 2 and 3 in (B) and lanes 3 and 4 in (C) represent elimination of a lane, which was run on the same gel but not relevant to the current study.

Figure 1 and Figure 2 demonstrate that α-toxin has immediate and prolonged effects on platelets. To explore prolonged responses in more detail, we screened for Bcl-3 synthesis in α-toxin stimulated platelets. Previous studies have shown that Bcl-3 is synthesized by thrombin-activated platelets, a response that requires engagement of α_IIb_β_3_ integrins on the surface of platelets [11,12,13,14]. Consistent with published work [11,14], Bcl-3 mRNA was detected in unactivated and α-toxin stimulated human platelets [Figure 3(A)]. In contrast, cultured platelets accumulated Bcl-3 protein, a response that was accentuated by α-toxin [Figure 3(B)]. Specifically, we observed trace amounts of Bcl-3 protein in platelets that were resuspended in M199 with soluble fibrinogen [lane 1; Figure 3(B)], which may be due to auto-activation of platelets during the four hour culture period. Thrombin or α-toxin accentuated Bcl-3 protein expression in platelets that were resuspended and cultured in the presence of soluble fibrinogen [lanes 2 and 3; Figure 3(B)]. The addition of leukocytes to platelet preparations did not enhance the expression of Bcl-3 protein (data not shown). These results, in combination with Figure 3(A) showing that α-toxin does not induce transcription of Bcl-3 mRNA, demonstrate that contaminating leukocytes do not contribute to increased Bcl-3 protein expression in these studies. 

Consistent with previous studies from our group [12], platelets adherent to immobilized fibrinogen or collagen accumulated Bcl-3 protein in a time-dependent fashion [Figure 3(C) and Supplemental Figure 2(A,B)]. Thrombin or α-toxin did not appreciably increase Bcl-3 protein expression in fibrinogen-adherent platelets [Figure 3(C) and Supplemental Figure 2(A)]. In contrast, Bcl-3 protein expression was markedly increased in collagen-adherent platelets that were co-incubated with α-toxin for four hours [Supplemental Figure 2(B)]. This increase, however, waned considerably after a 12 hour period [Supplemental Figure 2(B)].

**Figure 4:**
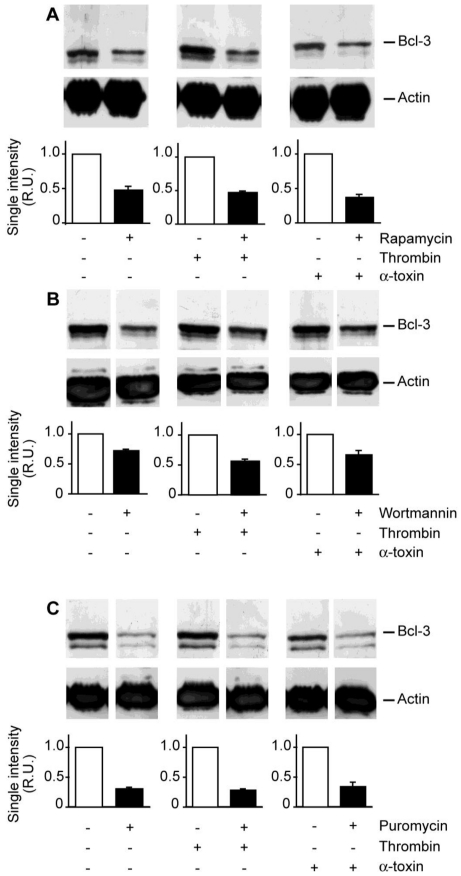
Accumulation of Bcl-3 protein in α-toxin stimulated platelets is reduced by protein synthesis inhibitors. Freshly-isolated platelets were cultured in suspension in the presence of rapamycin (**A**), wortmannin (**B**), or puromycin (**C**) for 30 minutes. After this incubation period, soluble fibrinogen was added to the culture and then the platelets were treated with vehicle, thrombin, or α-toxin for 240 minutes. Bcl-3 (top panels) or actin (bottom panels) protein expression was subsequently assessed by Western blot analysis. The gels in each figure are representative of five independent experiments. The bars below each gel display the average densitometry data for each experimental condition (mean ± SEM, n = 5). Note, all samples were run on the same gel but, in some cases (B and C), comparative samples were separated by extra lanes and therefore cropped individually.

We previously demonstrated that Bcl-3 synthesis is controlled by mTOR in thrombin-activated platelets [13,14]. To determine if α-toxin activates similar signaling pathways, we pretreated platelets with rapamycin or wortmannin. Rapamycin negates mTOR activity while wortmannin neutralizes the activity of phosphoinositide 3-kinase (PI3K), which is upstream of mTOR [14]. We found that both inhibitors attenuated Bcl-3 protein expression in α-toxin stimulated platelets [Figure 4(A,B)]. Puromycin, a global inhibitor of mRNA translation, also significantly reduced Bcl-3 protein expression in α-toxin-stimulated platelets [Figure 4(C)].

## 4. Discussion

Gram-positive *S. aureus* is the most frequent causative organism of infective endocarditis [16], a disease characterized by colonies of bacteria and platelets that reside on cardiac valves [3]. Strains of *S. aureus* that produce α-toxin are present in cardiac vegetations and induce experimental endocarditis [6]. Previous studies have also shown that specific components of *S. aureus*, such as clumping factor A and fibronectin binding protein A, regulate immediate responses of platelets including aggregation [22]. α-toxin rapidly activates platelets by integrating into platelet membranes and promoting blood coagulation [7,9], and intravenous administration of α-toxin in cynomolgus monkeys elicits a selective drop in platelets counts [23]. Here, we show that α-toxin also induces prolonged responses in platelets, including distinct morphologic signs of activation and synthesis of Bcl-3 protein. These types of activation events may exist in infective endocarditis where platelet and *S. aureus* vegetate with one another and α-toxin is continually secreted into the local milieu.

α-toxin forms pores in cellular membranes that facilitate an influx of extracellular Ca^2+^ [24]. Calcium fluxes contribute to α-toxin’s cytolytic effect on target cells and, over time, may contribute to the morphologic features observed in the present study. When suspension platelets were exposed to α-toxin for four hours, they were visibly activated. α-toxin stimulated platelets extended pseudopodia and cellular fragments were observed throughout the extracellular milieu. Another striking feature is the development of “string-like” interconnections between α-toxin stimulated platelets. It is possible that these strings are fibrin strands, which evolve as platelets retract clots. Previous studies from our group demonstrate that washed platelet preparations develop and retract fibrin strands when they are cultured and activated in the presence of soluble fibrinogen [13]. Another possibility is that these string-like structures are thin extensions of cell membrane, especially since they stain for sialic acids. They do not, however, stain for actin. Whether these strands are made of fibrin, fibrin that is laced by sialic acids, or are extensions of sialic-rich cell membranes requires further investigation. Nevertheless, these results suggest that α-toxin induces platelets to produce extracellular tethers that promote platelet-platelet interactions. This response, in combination with the generation of microparticle debris, may also contribute to the pro-coagulant activities of α-toxin on platelets [9]. 

Albeit different than responses in suspension cultures, α-toxin also induced morphologic changes in platelets that adhered to extracellular matrices. The most prominent feature is that adherent platelets distribute their granules to the cell periphery. This suggests that α-toxin stimulated platelets secrete their granule contents as they adhere to immobilized collagen or fibrinogen. Adherent platelets also coalesced polymerized actin, but they did not extend pseudopodia in response to α-toxin and, in fact, remained discoid and readily adhered to both extracellular matrices. These results indicate that platelets retain their capacity to adhere and mobilize intracellular granules in the presence of α-toxin, but suggest that α-toxin induces distinct morphologic changes in circulating *versus* adherent platelets. It is also possible that the adherent platelets initially extended, and then retracted, pseudopodia at earlier time points that were not captured by the current experimental parameters.

In addition to its effects on platelet morphology, α-toxin induced Bcl-3 protein synthesis by platelets. Bcl-3 is an oncogene that was unexpectedly identified in activated, but not resting, platelets [14]. We previously demonstrated that platelets express and translate mRNA for Bcl-3 in response to collagen or thrombin stimulation [12,14]. α-toxin adds to the repertoire of agonists that induce Bcl-3 synthesis by platelets and suggests that *S. aureus* and other bacterial strains may have similar effects. Indeed, Shashkin and colleagues [25] recently demonstrated that *E. Coli*-derived lipopolysaccharide induces the synthesis of interleukin-1β protein by platelets. Similar to thrombin stimulated platelets [13,14], the mTOR translational control pathway regulates α-toxin induced Bcl-3 protein synthesis. mTOR-dependent synthesis of Bcl-3 controls platelet-dependent clot retraction [13]. It is also likely that α-toxin stimulated platelets synthesize Bcl-3 to facilitate platelet-dependent clot retraction, especially since platelets condense and coalesce polymerized actin in response to α-toxin. 

Platelets synthesize Bcl-3 via integrin α_IIb_β_3_. In this regard, previous studies from our group showed that platelets from patients with Glanzmann Thrombasthenia, which lack integrin α_IIb_β_3_, do not synthesize Bcl-3 [12]. This, in combination with inhibitory antibody based studies, demonstrated that platelets rely on integrin α_IIb_β_3_ for Bcl-3 synthesis [12]. In the current study, α-toxin induces α_IIb_β_3_-dependent aggregation suggesting that platelets use similar outside-in signaling pathways to synthesize Bcl-3 when they are exposed to α-toxin. In support of this, suspension cultured platelets synthesized Bcl-3 in the presence of soluble fibrinogen, which enhances α_IIb_β_3_-dependent aggregation. As previously shown by our group [12] and confirmed here, we also found that platelets synthesized Bcl-3 after they adhered to immobilized fibrinogen. Adherence to immobilized fibrinogen directly activates integrin α_IIb_β_3_ [26]. α-toxin, however, did not accentuate Bcl-3 synthesis by fibrinogen-adherent platelets. α-toxin did increase Bcl-3 synthesis by platelets that adhered to collagen for four hours, but not longer time periods. The reasons for these differential synthetic responses between fibrinogen and collagen and the temporal variances in collagen-adherent platelets are not clear. Nonetheless, they demonstrate that multiple types of signaling events are capable of inducing Bcl-3 synthesis by platelets.

In summary, interactions between platelets and *S. aureus* are common in infective endocarditis and the results from the current study indicate that *S. aureus*-derived exotoxins induce immediate and prolonged activation responses in target platelets. α-toxin’s effects on platelet function may have adverse consequences during the development, evolution and resolution of endocarditis and other infectious situations.

## Figures and Tables

**Figure 1:**
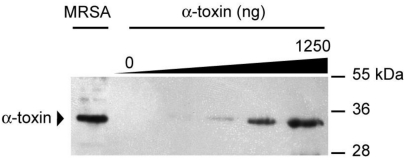
Methicillin Resistant Staphyloccal Aureus (MRSA) secretes α-toxin. MRSA (9 × 10^4^ CFU), which was isolated from a patient diagnosed with sepsis, was placed in M199 (1 mL) media for eight hours. A portion of the releasate (50 μL) was subsequently placed in an equal amount of SDS loading buffer. The releasate was separated by electrophoresis and probed with an antibody directed against α-toxin. Varying amounts of recombinant α-toxin (156 ng, 312 ng, 625 ng, and 1.25 μg) were run in parallel. This experiment is representative of two independent experiments.

**Figure 2:**
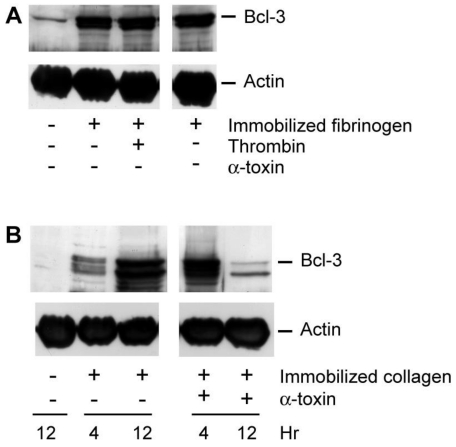
α-Toxin alters Bcl-3 protein accumulation in platelets adherent to collagen, but not fibrinogen. (**A**) Freshly-isolated platelets were cultured in suspension (lane 1) or adhered to immobilized fibrinogen for 12 hours in presence of vehicle (lane 2), thrombin (lane 3), or α-toxin (lane 4). The top and bottom panels display Bcl-3 and actin expression, respectively. The dividing line between lanes 3 and 4 represent elimination of a lane, which was run on the same gel but not relevant to the current study. The gel is representative of five independent experiments. (**B**) Freshly-isolated platelets were cultured in suspension (lane 1) or adhered to immobilized collagen for four hours in presence of vehicle (lane 2) or α-toxin (lane 4) or 12 hours in the presence of vehicle (lane 3) or α-toxin (lane 5). The top and bottom panels display Bcl-3 and actin expression, respectively. The dividing line between lanes 3 and 4 represent elimination of a lane, which was run on the same gel but not relevant to the current study. The gel is representative of four independent experiments.
